# Rapid set up of research centres in a trial in an evolving research governance world

**DOI:** 10.1186/1745-6215-14-S1-P132

**Published:** 2013-11-29

**Authors:** Sharon Ruddock, Bipin Bhakta, Amanda Lilley-Kelly, Suzanne Hartley, Lorna Barnard

**Affiliations:** 1 Leeds Institute of Clinical Trials Research, Leeds, UK; 2 Academic Department of Rehabilitation Medicine, Leeds, UK

## 

The Dopamine Augmented Rehabilitation in Stroke (DARS) trial is a double-blind placebo controlled trial investigating impact of co-careldopa/placebo in combination with routine NHS occupational/physical therapy on functional outcome in people with acute stroke. Recruitment to the trial was lower than anticipated and recruitment predictions suggested that we needed to increase the number of recruiting sites from 20 to at least 40 to achieve an acceptable recruitment rate. This needed to be done as soon as possible to impact on recruitment. We will present the strategic plan which was developed to ensure sites opened to recruitment in a short timeframe. Key components included: collaborating with the Stroke Research Network to identify centres which could adhered to the trial protocol, had a track record in obtaining NHS Permissions in a short timeframe and to provide local knowledge to resolve any issues; identifying and engaging with a dedicated contact at each centre to ensure timely identification of issues; recruitment of a dedicated trial co-ordinator to actively manage the set-up process at each site and the use of a central email account to support the influx of queries. Successful implementation of the plan required close collaboration between the local site staff, the SRN Managers and the central trials team. The plan meant that the average time from receiving Expressions of Interest to NHS Permissions was reduced to 4 months and the recruitment rate has steadily increased to an acceptable rate accordingly.

